# Clinical Profiles and Prognostic Patterns in Critically Ill Cardiac Patients Requiring Invasive Mechanical Ventilation: A Five-Year Retrospective Cohort Study

**DOI:** 10.3390/diagnostics16081237

**Published:** 2026-04-21

**Authors:** Liviu Macovei, Andreea Chiper, Daniel Dăscălescu, Cristian Stătescu, Grigore Tinică

**Affiliations:** 1 “Grigore T. Popa” University of Medicine and Pharmacy, 16 University Street, 700115 Iasi, Romania; 2 “Prof. Dr. George I.M. Georgescu” Institute of Cardiovascular Diseases, 50 Carol I Blvd., 700503 Iasi, Romania

**Keywords:** critically ill cardiac patient, invasive mechanical ventilation, prognostic patterns, in-hospital mortality

## Abstract

**Background:** Critically ill cardiac patients who require invasive mechanical ventilation represent a high-risk population with persistently elevated in-hospital mortality, despite advances in cardiovascular and critical care management. Real-world data describing clinical profiles and prognostic patterns in this population remain limited. **Objectives:** The aim of this study was to characterize clinical profiles and prognostic patterns among critically ill cardiac patients requiring invasive mechanical ventilation and to identify variables associated with in-hospital mortality. **Methods:** We conducted a five-year retrospective observational cohort study, including 492 adult patients admitted to a tertiary cardiovascular intensive care unit who required invasive mechanical ventilation. The demographic characteristics, cardiovascular risk factors, primary cardiac diagnoses, major in-hospital complications, duration of mechanical ventilation, length of hospital stay, and in-hospital mortality were analyzed. **Results:** The overall in-hospital mortality was 53.9%. Acute myocardial infarction was the most frequent primary diagnosis. Advanced age, diabetes mellitus, cardiogenic shock, acute renal dysfunction, hepatic dysfunction and prolonged hospitalization were significantly associated with increased mortality (*p* < 0.05 for all comparisons). Cardiogenic shock showed the strongest association (*p* < 0.001). Ventilator-associated respiratory infections occurred in 16.9% of patients, and were associated with a prolonged hospital stay (*p* < 0.05), without a statistically significant association with mortality. **Conclusions:** Critically ill cardiac patients requiring invasive mechanical ventilation exhibit distinct high-risk clinical profiles characterized by advanced age, cardiogenic shock, metabolic comorbidities, and the development of multi-organ dysfunction. These findings highlight prognostic patterns that may support risk stratification and generate hypotheses for future prospective studies in cardiac intensive care.

## 1. Introduction

Cardiovascular diseases remain the leading cause of morbidity and mortality worldwide, with an increasing number of patients presenting with acute, life-threatening cardiac conditions that require admission to specialized intensive care units. Despite major advances in reperfusion strategies, pharmacological therapies, and mechanical circulatory support, critically ill cardiac patients continue to experience high short-term mortality and complex clinical trajectories.

The management of critically ill cardiac patients is currently performed in dedicated cardiac intensive care units, where multidisciplinary teams integrate cardiology, critical care medicine, advanced imaging, and invasive therapies. Although the organization and technological capabilities of cardiac intensive care units have evolved substantially over recent decades, mortality among the most severe patient subgroups remains unacceptably high, particularly in those requiring invasive organ support.

Invasive mechanical ventilation is frequently required in critically ill cardiac patients presenting with acute myocardial infarction complicated by cardiogenic shock, acute pulmonary edema, malignant arrhythmias, or cardiac arrest. While mechanical ventilation may be life-saving by reducing the respiratory workload and improving systemic oxygen delivery, it also exerts significant hemodynamic effects and may contribute to the development of secondary organ dysfunction, particularly in patients with a limited cardiovascular reserve [1,2]. The interaction between positive pressure ventilation and cardiovascular function is complex and may exacerbate circulatory instability in vulnerable patients. As a result, the decision to initiate invasive mechanical ventilation in cardiac intensive care requires careful clinical judgment, balancing potential life-saving benefits against the risk of hemodynamic deterioration and subsequent multi-organ failure [1,2].

Despite its clinical importance, the population of critically ill cardiac patients requiring invasive mechanical ventilation remains heterogeneous and insufficiently characterized in real-world settings. The existing prognostic scores used in general intensive care populations may not fully capture the cardiac-specific determinants of the outcome, underscoring the need for dedicated analyses focusing on this high-risk subgroup [1,2].

In this context, retrospective cohort studies may provide valuable insights by identifying the clinical profiles and prognostic patterns associated with adverse outcomes. Such analyses can generate hypotheses for future prospective research and support improved risk stratification and clinical decision-making in cardiac intensive care [1,2].

## 2. Materials and Methods

### 2.1. Study Design and Setting

This study is a retrospective, observational cohort analysis conducted in the cardiac intensive care unit of the “Prof. Dr. George I.M. Georgescu” Institute of Cardiovascular Diseases, Iași, Romania. The study period spanned five consecutive years and included all eligible patients who required invasive mechanical ventilation for acute cardiac conditions during hospitalization.

### 2.2. Study Population

The study population consisted of adult patients (≥18 years) admitted to the cardiac intensive care unit who required invasive mechanical ventilation via orotracheal intubation as part of the management of an acute, life-threatening cardiac condition.

Invasive mechanical ventilation was defined as ventilatory support delivered through an endotracheal tube following emergency or urgent orotracheal intubation. Patients treated exclusively with non-invasive ventilation were not included.

### 2.3. Inclusion and Exclusion Criteria

The inclusion criteria were: (1) admission to the cardiac intensive care unit for an acute cardiovascular condition; (2) a requirement for invasive mechanical ventilation; and (3) the availability of complete clinical and outcome data during the index hospitalization.

The exclusion criteria included: (1) admission for primary surgical emergencies; (2) terminal non-cardiac illness with palliative intent at admission; and (3) incomplete medical records that precluded reliable data extraction.

### 2.4. Data Collection

Clinical data were collected retrospectively from electronic and paper-based medical records. The extracted variables included the demographic characteristics, cardiovascular risk factors, primary cardiac diagnosis at admission, major in-hospital complications, duration of invasive mechanical ventilation, length of hospital stay, and in-hospital mortality. Comorbidities were defined according to the documented medical history at admission and included diabetes mellitus, arterial hypertension, chronic kidney disease, dyslipidemia, and obesity.

### 2.5. Definitions of Key Clinical Conditions

Acute myocardial infarction was defined according to the Fourth Universal Definition of Myocardial Infarction as a rise in cardiac troponin values with at least one value above the 99th percentile upper reference limit, in the presence of clinical and paraclinical evidence of myocardial ischemia [3].

Cardiogenic shock was defined, according to contemporary clinical criteria, as being persistent hypotension (systolic blood pressure <90 mmHg or the need for vasopressor therapy to maintain a mean arterial pressure ≥65 mmHg), associated with clinical signs of end-organ hypoperfusion, in the setting of primary cardiac dysfunction [4,5,6].

Due to the retrospective nature of the study, advanced hemodynamic parameters (e.g., cardiac index, pulmonary artery pressures), serum lactate levels, and standardized shock severity scores were not uniformly available and were therefore not included in the primary analysis.

### 2.6. Definition of Acute Organ Dysfunction

Acute kidney injury was defined according to the KDIGO criteria. Acute respiratory distress syndrome was defined according to the Berlin definition. Sepsis was defined according to the Sepsis-3 criteria, where applicable [7,8,9].

Acute renal dysfunction was defined as either the development of acute kidney injury during hospitalization or an acute exacerbation of pre-existing chronic kidney disease, as documented in medical records. Hepatic dysfunction was defined by clinical diagnosis and laboratory abnormalities that were consistent with acute or chronic liver failure occurring during hospitalization. Neurological dysfunction included documented hypoxic–ischemic encephalopathy following cardiac arrest or prolonged hypotension [10].

### 2.7. Infectious Complications

Respiratory tract infections were defined as ventilator-associated respiratory infections occurring more than 48 h after the initiation of invasive mechanical ventilation, based on clinical, microbiological, and radiological criteria [11,12,13].

### 2.8. Ethical Considerations

This retrospective study was conducted in accordance with the Declaration of Helsinki. In compliance with institutional regulations, informed consent for medical procedures and for the use of anonymized clinical data for academic and scientific purposes was obtained at hospital admission from patients or their legally authorized representatives. The study protocol was reviewed and approved by the Institutional Review Board of the “Prof. Dr. George I.M. Georgescu” Institute of Cardiovascular Diseases, Iași, Romania (approval no. 3354/11.11.2025).

### 2.9. Statistical Analysis

Statistical analysis was performed using SPSS version 25 (IBM Corp., Armonk, NY, USA). Continuous variables are presented as mean ± standard deviation, while categorical variables are expressed as absolute numbers and percentages.

Comparisons between survivors and non-survivors were performed using Student’s *t*-test or the Mann–Whitney U test for continuous variables and Chi-square or Fisher’s exact test for categorical variables. A *p*-value < 0.05 was considered statistically significant. Given the retrospective and exploratory nature of the study, analyses were intended to identify clinically relevant associations and generate hypotheses for future prospective research without implying causal relationships.

## 3. Results

### 3.1. Baseline Characteristics

The study cohort included 492 critically ill cardiac patients requiring invasive mechanical ventilation. Of these, 313 patients (63.6%) were male and 179 (36.4%) were female. The mean age was 68.1 ± 13.2 years (range 18–98 years). Most patients were older than 60 years (*n* = 281), and this age group accounted for the majority of in-hospital deaths (*p* < 0.001).

### 3.2. Cardiovascular Risk Factors and Comorbidities

At least one major cardiovascular risk factor was present in 308 patients (63.0%), while 145 patients (29.5%) had two or more risk factors. Diabetes mellitus was documented in 167 patients (33.9%) and was significantly associated with in-hospital mortality (*p* = 0.03). Arterial hypertension, dyslipidemia, and obesity were frequently observed but did not show a significant association with mortality in univariate analysis. Chronic kidney disease at admission was present in 109 patients (22.2%) and was not independently associated with mortality. The baseline characteristics and cardiovascular risk factors are presented in Table 1.

### 3.3. Primary Cardiac Diagnosis and Indication for Mechanical Ventilation

Acute myocardial infarction represented the most frequent primary diagnosis, occurring in 373 patients (75.8%). Anterior wall myocardial infarction was the most common localization. Infarct territory was not significantly associated with in-hospital mortality.

Cardiogenic shock was present in 218 patients (44.3%) and was strongly associated with in-hospital mortality (*p* < 0.001), particularly among patients older than 60 years.

Acute pulmonary edema requiring invasive mechanical ventilation was documented in 149 patients (30.3%) and was not independently associated with mortality.

### 3.4. Duration of Mechanical Ventilation and Length of Hospital Stay

The mean length of hospital stay was 10.0 ± 9.1 days. Regarding invasive mechanical ventilation, 155 patients (31.5%) required ventilatory support for less than 24 h, 154 patients (31.3%) for 24–96 h, and 183 patients (37.2%) for more than 96 h. Prolonged mechanical ventilation was associated with increased morbidity and a higher incidence of in-hospital complications.

Furthermore, the death rate for mechanically ventilated intubated critical care cardiac patients was significantly higher in the first 10 hospitalization days (*p* < 0.001) (Table 2).

### 3.5. Acute Organ Dysfunction

1. Acute myocardial infarction

**Figure 1:**
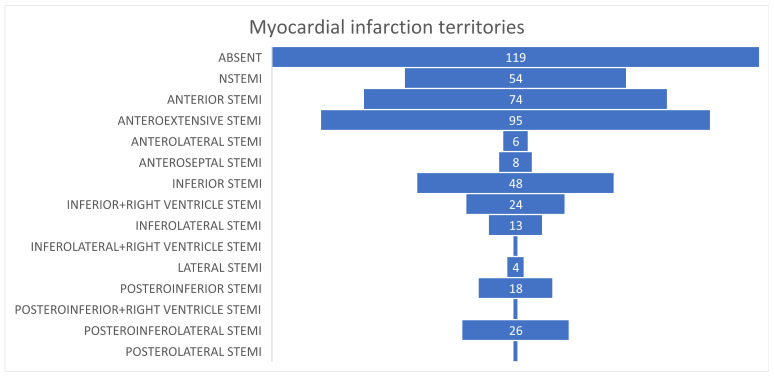
Territory affected by myocardial infarction.

A total of 373 of the patients presented various clinical presentations of acute myocardial infarction at admission: most frequently, the localization of infarction being in the anterior territory. Figure 1 shows the number of subjects for each territory affected by STEMI. However, the STEMI location does not represent a significant death predictor. Age represents an important prognostic factor: 281 patients found themselves in the over 60 years old category and only 92 were under 60. Also, we observed a higher mortality rate in the over 60 years old patients, with 163 having a fatal evolution; meanwhile, in the under 60 age group, the mortality rate was equal to the survival one.

The territory affected by myocardial infarction did not correlate significantly to the risk of death (Table 3).

2. Cardiogenic shock

A total of 218 of the patients that required intubation and mechanical ventilation presented with cardiogenic shock (44.3%). The importance of this pathology can be confirmed by studying the number of deaths occurring in these patients. In the over 60 age group, of167 cases, 124 culminated with exitus, and in the under 60 age group, of the 51 patients with cardiogenic shock, 37 died (Table 4).

The presence of cardiogenic shock was associated with a significantly higher mortality in intubated and mechanically ventilated patients with *p* < 0.001 (Table 5).

3. Acute pulmonary edema

Acute pulmonary edema represents another relatively frequent cause of orotracheal intubation and mechanical ventilation (Table 6).

Acute pulmonary edema of cardiac origin occurs secondary to a rapid rise in pulmonary capillaries’ hydrostatic pressure, and is most likely found in pathologies with left ventricle dysfunction such as acute myocardial infarction and valvular dysfunctions, but also in the case of the occurrence of rhythm disorders such as ventricular tachycardia and total or high-grade atrio-ventricular blocks. Acute pulmonary edema refractory to medication (that persists over 15 min) requires orotracheal intubation and mechanical ventilation. However, acute pulmonary edema as a cause of orotracheal intubation and mechanical ventilation does not represent an ulterior predictor for death (*p* = 0.96) (Table 7).

4. Other organ dysfunction

During hospitalization, acute renal dysfunction developed in 286 patients (58.2%). The development of acute renal dysfunction was significantly associated with increased mortality (*p* < 0.05). Hepatic dysfunction occurred in 187 patients (38.0%) and was also significantly associated with adverse outcomes (*p* < 0.05). Neurological complications, including hypoxic–ischemic encephalopathy, were documented in 36 patients (7.3%) and were associated with poor prognosis.

### 3.6. Infectious Complications

#### Acute Respiratory Infection

An important complication in intubated and mechanically ventilated cardiac ill patients are respiratory tract infections, which require specific antimicrobial treatment. These infections that are trivial in appearance can have devastating effects in critical care cardiac ill patients with fulminant evolution to septic shock. A total of 16.9% of the analyzed patients, representing 83 subjects, developed a respiratory infection during their stay; the pathogens found included *Pseudomonas aeruginosa*, *Klebsiella pneumoniae*, and *Acinetobacterspp*, which are nosocomial agents that are multidrug resistant (Table 8). Ventilator-associated respiratory infections occurred in 83 patients (16.9%). Among these patients, 38 progressed toward death. The occurrence of respiratory infections was associated with prolonged hospitalization compared with the overall cohort (*p* < 0.05), without a statistically significant impact on mortality (*p* = 0.21).

Of the 83 patients with respiratory infections, 38 progressed toward death. The mean hospital stay in ventilated patients with a respiratory infection was roughly four days longer than the general mean (Table 9).

### 3.7. Cardiac Rhythm Disturbances

Severe cardiac rhythm disturbances were frequently observed. Sustained ventricular tachycardia occurred in 109 patients (22.2%), ventricular fibrillation in 104 patients (21.1%), and asystole in 164 patients (33.3%). A total atrioventricular block was documented in 71 patients (14.4%). These rhythm disturbances were commonly observed in patients with cardiogenic shock and multi-organ dysfunction.

### 3.8. Left Ventricle Systolic Dysfunction

The ejection fraction represents the most important evaluation parameter for systolic dysfunction of the left ventricle (Table 10). Also, an element of importance that influenced left ventricle insufficiency is represented by myocardial revascularization through coronary angioplasty. During hospitalization, 214 patients (43.5%) were subjected to a minimally invasive intervention of a percutaneous coronary angioplasty with a stent.

However, the decrease in the ejection fraction at admission was not significantly associated (*p* < 0.368) with the mortality of the studied patients, with *p* < 0.368.

### 3.9. In-Hospital Mortality

The overall in-hospital mortality was 53.9% (265 of 492 patients). Mortality was influenced by the cumulative burden of clinical severity, rather than a single isolated factor. Mortality was higher among older patients and in those with cardiogenic shock, diabetes mellitus, acute renal dysfunction, hepatic failure, and prolonged hospitalization (Table 11). No single variable alone explained the mortality risk; rather, adverse outcomes were associated with the accumulation of clinical severity markers and the development of multi-organ dysfunction.

Of all the deaths, 103 patients (38.9%) were female and 162 (61.1%) were male, with a *p* value equal to 0.216, not registering a statistical significance; this aspect was probably due to the fact that the male population included in our study was way larger than the female one.

### 3.10. The Role of Comorbidities in Intubated and Mechanically Ventilated Ill Patients’ Mortality

In our study, diabetes represented a significant predictor of death: of 167 diabetic patients, 106 (63.5%) died, with a *p* value < 0.002.

However, we did not register significant differences in the number of deaths for patients with or without arterial hypertension.

An old diagnosis of chronic kidney disease did not significantly influence the number of deaths (Table 12).

However, when we studied the total patients that developed renal dysfunction during hospitalization (either a flare of chronic kidney disease or a newly installed acute kidney failure), we obtained a *p* value < 0.001, which was statistically significant (Table 13). This way, we can affirm that, even though chronic kidney disease on its own does not influence mortality, the development of an acute kidney injury significantly increases the death rate in intubated and mechanically ventilated cardiac patients.

Liver failure that developed during hospitalization or was preexisting is associated with a significantly higher mortality rate in intubated and mechanically ventilated ill cardiac patients (Table 14).

## 4. Discussion

This retrospective cohort study describes clinical profiles and prognostic patterns in critically ill cardiac patients requiring invasive mechanical ventilation in a tertiary cardiac intensive care unit. The results highlight the high mortality of this population and underline the complexity of managing patients with severe cardiac disease that is complicated by respiratory failure and multi-organ dysfunction.

Critically ill cardiac patients represent a heterogeneous group characterized by advanced age, a high burden of comorbidities, and severe acute cardiac events. In the present cohort, acute myocardial infarction was the most frequent primary diagnosis, and cardiogenic shock emerged as a key clinical condition associated with adverse outcomes, particularly among older patients [1,2].

Among the most common causes of admission to emergency services in the United States is acute heart failure, with one in five patients being transferred to an intensive care unit and, moreover, of the number of admissions for cardiogenic shock due to myocardial infarction, 31.9% were complicated by multiple organ dysfunction. From a physiopathological point of view, heart failure is a syndrome resulting from any structural or functional dysfunction that affects the ability of the ventricles to fill or eject blood. The AHA recommends an initial direct etiological approach according to the acronym CHAMP (C—acute coronary syndrome, H—hypertensive emergencies, A—arrhythmias, M—acute mechanical causes, and P—pulmonary thromboembolism). Subsequently, after excluding these etiologies that may require life-saving interventions, the physician should continue the clinical and paraclinical evaluation (detailed medical and family history, complete clinical examination, cardiac and non-cardiac imaging, laboratory tests), diagnosing possibly less urgent causes [1,14].

One of the cardiovascular emergencies that affects millions of patients worldwide, with a prevalence of 9.5% in the population over 65 years of age, is acute myocardial infarction [1,14].

Thus, of the 492 patients in our study who required continuous ventilatory support, a total of 373 patients (75.8%) presented with some form of acute coronary syndrome; 281 were over 60 years old, with 163 deaths in the same age group, highlighting the importance of the age of the patients in the occurrence and evolution of this pathology.

Despite the implementation of revascularization therapy for acute myocardial infarction, the development of therapies for STEMI, and the advancement of technologies in mechanical circulatory support devices, the short-term prognosis of patients in cardiogenic shock remains poor, with in-hospital mortality rates being between 40 and 50%. Observational studies conducted recently suggest that more than half of the causes of cardiogenic shock are non-ischemic. In a study published by the AHA in June 2022, it was shown that of the 520 patients included (219 with cardiogenic shock after acute myocardial infarction and 301 with cardiogenic shock secondary to non-ischemic heart failure), a total of 158 patients died during hospitalization (30.4%) [14]. In comparison, patients with cardiogenic shock secondary to nonischemic heart failure were younger (mean age 58.5 years versus 65.6 years for patients with AMI), less likely to have comorbidities such as diabetes, and less likely to have cardiac arrest either at presentation or during hospitalization (15.9% versus 35.2%). Patients with nonischemic heart failure had lower left ventricular ejection fraction and were less likely to require vasopressor therapy upon admission. Of the total 520 patients, 272 required temporary mechanical circulatory support, with the most commonly used device being the intra-aortic balloon counterpulsation, and for patients with shock requiring biventricular or right ventricular support, veno-arterial ECMO was used most often. Regarding in-hospital mortality, the percentages were lower for patients with shock due to non-ischemic heart failure compared to that due to acute myocardial infarction. Also, one-year mortality was lower in patients with non-ischemic heart failure compared to those after acute myocardial infarction: this was 42.6% in the first category, as opposed to a mortality of 52.9% in the second one [14].

Also, in our study, cardiogenic shock was a condition with high mortality, with the most common etiology of shock being acute myocardial infarction. In this case too, the number of patients was significantly higher, with a total of 167 cases in the age group over 60 years old and only 51 in the patients under 60 years old, with a number of deaths of 124 and 37, respectively.

Cardiogenic shock is in about 5–7% of patients with AMI associated with 30-day high mortality—40–50% [4,5]. In patients with AMI and CS, use of early revascularization may reverse the hemodynamic damage and pump failure [6,15]. The patients with AMI and CS typically have high filling pressure, biventricular failure and secondary pulmonary hypertension, resulting in decreased gas exchange and increased work of breathing, contributing to acute respiratory failure [6,16,17]. The high metabolic demand from the increased work of breathing altered the mental status, resulting in pure synchrony, concomitant cardiac arrest and severity of pulmonary edema with poor medical response, causing insufficient oxygenation, acidosis, and an increase in lactate, all of which require tracheal intubation and the use of mechanical ventilation [6,18]. Another common indication for ventilation is to establish a safe airway in comatose patients, including patients with out-of-hospital arrest which comprise up to 50% of AMI and CS [4,19,20].

The use of mechanical ventilation was noted in 43.2% of 439,436 patients with AMI and CS during 2000–2014 in USA, which was significantly lower than in the CardShock and LABP-SCHOCK 2 trials [6].The optimal timing of intubation in AMI and CS is early timing. In this setting, the respiratory distress and cardiac output distributed to the respiratory muscles may increase by up to 10-fold, compared to healthy individuals. By decreasing the respiratory workload, early mechanical ventilation may improve oxygen delivery to vital organs [4,21]. A post hoc analysis of the CULPRIT SCHOCK trial demonstrated a 10% lower 1-year mortality in AMI and CS patients ventilated upon hospital admission compared to late intubation [22]. In the same direction, van Diepen et al. have revealed an association between 30-day mortality and each 1hour delay in the intubation of patients with refractory cardiogenic shock [4,23]. Mechanical ventilation has primarily favorable effects on the cardiac index and pulmonary capillary wedge pressure [4,24].

In our study, several clinical variables were associated with increased in-hospital mortality, including old age, diabetes mellitus, acute renal dysfunction, hepatic failure, and prolonged hospitalization. Importantly, the development of acute organ dysfunction during hospitalization, rather than the presence of chronic comorbidities alone, was strongly associated with adverse outcomes.

These findings support the concept that mortality in critically ill cardiac patients requiring invasive mechanical ventilation is driven by the accumulation of severity markers and the progression toward multi-organ dysfunction, rather than by a single isolated clinical factor.

Older age, a high degree of previous disability, and a greater range of comorbidities appear to be the most important predictors of mortality in patients that are mechanically ventilated for long periods [25]. In a study made in the USA during 2000–2014, of 439,436 patients with AMI and CS, 60% were male and non-white [6].

In our study, the number of critically ill male cardiac patients that were intubated and mechanically ventilated is almost double the number of female patients. The majority of patients included in our study (63%) had one or more risk factors: 33% had a single factor and 30% had two or more factors. Thus, 33.9% of patients were diabetic. According to the International Diabetes Federation, the prevalence of diabetes mellitus worldwide in the 20–79 age group in the general population is 10.5%; therefore, only two-thirds of the values were obtained in the study [26]. Also, 22.2% had a diagnosis of chronic kidney disease, 17.1% had hypertension, and 24.6% had some form of dyslipidemia.

A critical cardiac event can evolve with the dysfunction of several organs: at a renal level by decreasing renal perfusion; at a central nervous system level by cerebral hypoperfusion, which causes hypoxic encephalopathy; and at a hepatic level (liver failure) and gastrointestinal level (mesenteric ischemia). Multiorgan failure has been recognized as a significant contributor to morbidity and mortality [6,10].

The data from our study revealed a rate of development of acute renal dysfunction of 58.2% (36% acute renal failure and 22.2% exacerbation of a pre-existing chronic kidney disease). At the level of other systems, liver failure was detected in 38% of cases, 5.3% presented an episode of upper digestive bleeding and 7.3% of cases were complicated with hypoxic encephalopathy.

Ventilator-associated respiratory infections occurred in a substantial proportion of patients and were associated with prolonged hospitalization. Although not independently predictive of mortality in this cohort, these infections represent an important source of morbidity and resource utilization in cardiac intensive care units. In another study, nosocomial infection of the lung parenchyma impacted 20–36% of critically ill patients [11,12,13]. Mortality rates for ventilator-associated pneumonia span a wide range—24–76% [11,27,28]. High rates of pathogen resistance have emerged in patients with ventilator-associated pneumonia, making a therapeutic choice challenging. For the progress in pathogen detection, use of new biomarkers to guide treatment and new antibiotic association and delivery devices can improve the prognosis of ventilator-associated pneumonia [11,29,30].

In our study, 16.87% of patients developed a respiratory tract infection associated with mechanical ventilation, which is a value that is comparable to the one detected worldwide. Compliance with hygiene standards and the prevention of nosocomial infections are important things to consider when talking about reducing the rate of infections associated with the use of continuous ventilatory support [31]. The clinical pulmonary infection score was created to predict the pretest probability of nosocomial pneumonia. This score combines information on body temperature, volume of tracheal secretions, chest radiograph findings, white blood cell count, oxygenation and tracheal aspirate culture [29]. Clinical examination has a sensitivity of 66.4% and specificity of 53.9% [11]. Nosocomial infections are often caused by ESKAPE pathogens: *Enterococcus faecium*, *Staphylococcus aureus*, *Klebsiella pneumoniae*, *Acinetobacter baumannii*, *Pseudomonas aeruginosa*, *Enterobacter* spp. [29,32].

In a study published in 2022 by Davodian L.W et al., which included 1716 patients with AMI and CS, the cause of death was mainly heart failure followed by neurologic injury and MOF. The median time to death was 10 times longer in patients dying from neurologic injury or MOF compared with only 13 h in patients dying from primary heart failure [33]. In cases of death due to cardiac causes, some will die from refractory arrhythmias, some because of mechanical complications and some because of refractory pump failure. The patients from this latter group will invariably develop MOF [33,34,35]. In a very important study published by Vallabhajosyula S et al., the mortality rate for the cohort with acute respiratory failure and mechanical ventilation was 40%, which is similar to the data from the CardShock Registry [6,17].

Overall, the observed in-hospital mortality of 53.9% reflects the extreme severity of illness in our population. The present analysis emphasizes that critically ill cardiac patients requiring invasive mechanical ventilation constitute a distinct high-risk subgroup within cardiac intensive care, for whom early recognition of adverse prognostic patterns may be clinically relevant. Thus, we detected a significant statistical significance of the relationship between diabetes mellitus and arterial hypertension in relation to in-hospital mortality. Regarding chronic kidney disease, no significant relationship was detected between this and the death rate. However, a statistically significant relationship was obtained between the development of acute renal dysfunction and mortality, thus confirming the importance of renal pathology in cardiovascular impairment. Severe liver failure was associated with significantly more deaths. The diagnosis of cardiogenic shock was also statistically significant, with a good portion of patients who developed this condition progressing towards death.

We identify, in terms of the number of days of hospitalization, that the prognosis of intubated and mechanically ventilated patients becomes more severe as their number increases. Studies have shown an increase in morbidity and mortality in patients who are on long-term mechanical ventilation. A number of risk factors that are relevant to prolonged mechanical ventilation (mechanical ventilation for more than 21 days) have been identified, including age, the presence of comorbidities (previous stroke, cardiac dysfunction, renal impairment, COPD), and variations in biochemical parameters (low platelet count, elevated BUN, elevated creatinine, hypernatremia, hyperglycemia, low serum albumin). Blood gas values (low bicarbonate and pH levels or elevated PaCO_2_ levels), as well as ventilator parameters (FiO_2_ above 0.39) or gas exchange (PaO_2_/FiO_2_ ratio below 200 mm Hg) are also relevant in these patients. Risk calculation scores have also been developed; one of these is the I-TRACH, which is composed of six elements, namely, intubation in the intensive care unit (I); tachycardia, with a heart rate of more than 110 beats per minute (T); renal dysfunction, with BUN values over 25 (R); acidosis (A); creatinine values over 2 mg/dL or an increase in more than 50% of the initial value (C); and low serum bicarbonate values (H). Another element that leads to increased mortality in ventilated patients is the failure to disconnect from the ventilation system, with the risk factors in this case being old age, female gender, body mass index values that are too low or too high, the presence of COPD, neuromuscular diseases or hypertension [36,37,38,39].

## 5. Study Limitations

This study has several limitations that are inherent to its retrospective observational design. First, the analysis was based on data that were available in medical records, which limited the inclusion of certain variables such as standardized severity scores, advanced hemodynamic measurements, and selected biomarkers. Second, the single-center design may limit the generalizability of the findings. Third, no multivariable modeling was performed, and therefore no causal inferences can be drawn. The absence of multivariable analysis prevents the identification of independent predictors of mortality. Finally, the long-term outcomes after hospital discharge were not available.

## 6. Conclusions

Critically ill cardiac patients requiring invasive mechanical ventilation represent a distinct, extremely high-risk population in cardiac intensive care. In this five-year retrospective cohort, in-hospital mortality was substantial and was associated with advanced age, cardiogenic shock, diabetes mellitus, the development of acute renal and hepatic dysfunction, and prolonged hospitalization.

The findings indicate that adverse outcomes are related to the accumulation of clinical severity markers and the progression toward multi-organ dysfunction, rather than to a single isolated clinical factor. Acute organ dysfunction developing during hospitalization appears to play a more important prognostic role than pre-existing chronic comorbidities alone.

By describing clinical profiles and prognostic patterns in a real-world cohort of mechanically ventilated cardiac patients, this study provides hypothesis-generating data that may support improved risk stratification and inform the design of future prospective studies in cardiac intensive care.

The identified associations should be interpreted with caution due to the absence of multivariable modeling and should be considered hypothesis-generating, requiring validation in large prospective studies.

## Figures and Tables

**Table 1:** Baseline characteristics and cardiovascular risk factors (*n* = 492).

Parameter		Value	*p*-Value
**Sex distribution**	Male	313 (63.6%)	NS
	Female	179 (36.4%)	NS
**Age**	Mean age (years)	68.1 ± 13.2	
	Age ≥ 60 years	281 (57.1%)	***p*** **< 0.05**
	Age < 60 years	211 (42.9%)	NS
**Individual risk factors:**			
	Diabetes mellitus	167 (33.9%)	***p*** **< 0.002**
	Arterial hypertension	84 (17.07%)	NS
	Dyslipidemia	60 (12.19%)	NS
	Obesity	37 (7.52%)	NS
	Smoking	32 (6.5%)	NS
	Chronic kidney disease (baseline)	109 (22.2%)	***p*** ** = 0.419**
	Renal dysfunction (acute/worsening)	286 (58.1%)	***p*** **< 0.001**

**Table 2:** Distribution of deaths according to number of hospitalization days.

	Deceased	Survivors	No. of Patients	Statistical Significance
<10 days	183 (62.67%)	109 (37.33%)	292	***p*** **< 0.001**
10–30 days	75 (39.26%)	116 (60.73%)	191	
>30 days	7 (77.77%)	2 (22.23%)	9	
**Total**	265	227	**492**	

**Table 3:** Influence of affected territory over death rate.

Results				Statistical Significance
	**Deceased**	**Survivors**	**Total**	***p* = 0.1965 **
TOTAL STEMI	181 (56.73%)	138 (43.26%)	319	
ANTERIOR STEMI	112 (61.21%)	71 (38.79%)	183	
INFERIOR STEMI	25 (52.08%)	23 (47.91%)	48	
INFERIOR+ RIGHT VENTRICLE STEMI	9 (37.5%)	15 (62.5%)	24	
NSTEMI	28 (51.85%)	26 (48.15%)	54	

**Table 4:** Distribution of deaths according to age category of intubated and mechanically ventilated patients with cardiogenic shock.

	<60 Years	≥60 Years
**Deceased**	37	124
**Survivors**	14	43
**Total**	51	167

**Table 5:** Death rate in patients with cardiogenic shock.

		Cardiogenic Shock	No. of Patients	Statistical Significance
Deceased	YES	161 (60.75%)	265	
	NO	57 (25.11%)	227	***p* < 0.001**
Total		218	492	

**Table 6:** Acute pulmonary edema with intubation and mechanical ventilation need.

	N	%
**YES**	**149**	**30.3%**
NO	343	69.7%

**Table 7:** Distribution of deaths according to presence of acute pulmonary edema.

		Acute Pulmonary Edema	No. of Patients	Statistical Significance
Deceased	YES	80 (30.18%)	265	
	NO	69 (46.31%)	227	***p* = 0.960**
Total		149	492	

**Table 8:** Distribution of patients according to presence/absence of respiratory infection due to orotracheal intubation.

	No. of Patients	%
ABSENT	409	83.1%
PRESENT	83	16.9%

**Table 9:** Mean of hospitalization days according to deceased status of patients with respiratory infection.

Deceased	No. of Patients	Mean of Hospitalization Days
**Yes**	38	14.2
**No**	45	14.02

**Table 10:** Distribution of patients, according to left ventricle ejection fraction decrease.

Normal		Mildly Decreased	Moderately Decreased	Severely Decreased
**No. of patients**	396	20	33	43
**%**	80.50%	4.00%	6.70%	8.70%

**Table 11:** Distribution of deaths according to age category in patients with myocardial infarction.

	**<60 Years**	**≥60 Years**
**Deceased**	46 (12.33%)	163 (43.70%)
**Survivors**	46 (12.33%)	118 (31.63%)
**Total**	92	281

**Table 12:** Distribution of studied population according to death rate in chronic kidney disease patients.

		Chronic Kidney Disease	No. of Patients	Statistical Significance
Deceased	YES	55	265	
	NO	54	227	*p* = 0.419
Total		109	492	

**Table 13:** Renal dysfunction—new and flares of chronic kidney disease.

		Presence of Renal Dysfunction	No. of Patients	Statistical Significance
Deceased	YES	173	265	***p* < 0.001**
	NO	113	227	
Total		286	492	

**Table 14:** Distribution of deaths according to presence of hepatic failure.

		Hepatic Failure	No. of Patients	Statistical Significance
Deceased	YES	122	265	
	NO	65	227	***p* < 0.001**
Total		187	492	

## Data Availability

The data presented in this study are available upon request from the corresponding author, due to privacy and ethical restrictions.

## Abbreviations

The following abbreviations are used in this manuscript:

AMI	acute myocardial infarction
AHA	American Heart Association
BUN	blood urea nitrogen
CCU	coronary care unit
COPD	chronic obstructive pulmonary disease
CS	cardiogenic shock
ECMO	extracorporeal membrane oxygenation
MOF	multiple organ failure
NSTEMI	non-ST-elevation myocardial infarction
STEMI	ST-elevation myocardial infarction
